# Diabetes, metformin use, and survival in esophageal cancer: a population-based cohort study

**DOI:** 10.1093/jncics/pkad043

**Published:** 2023-06-14

**Authors:** Qiao-Li Wang, Giola Santoni, Jesper Lagergren

**Affiliations:** Upper Gastrointestinal Surgery, Department of Molecular Medicine and Surgery, Karolinska Institutet, Karolinska University Hospital, Stockholm, Sweden; Department of Medical Oncology, Dana-Farber Cancer Institute, Harvard Medical School, Boston, MA, USA; Upper Gastrointestinal Surgery, Department of Molecular Medicine and Surgery, Karolinska Institutet, Karolinska University Hospital, Stockholm, Sweden; Upper Gastrointestinal Surgery, Department of Molecular Medicine and Surgery, Karolinska Institutet, Karolinska University Hospital, Stockholm, Sweden; School of Cancer and Pharmaceutical Sciences, King’s College London, London, UK

## Abstract

**Background:**

It is unclear how diabetes and metformin use is associated with survival of esophageal cancer.

**Methods:**

This population-based cohort study included new cases of esophageal cancer reported in Sweden from 2006 to 2018 with follow-up through 2019. Diabetes status and metformin use were analyzed in relation to all-cause and disease-specific mortality using multivariable Cox regression. The hazard ratios (HRs) with 95% confidence intervals (CIs) were adjusted for age, sex, calendar year, obesity, comorbidity, and use of nonsteroidal anti-inflammatory drugs or statins. For comparison reasons, 3 other antidiabetic medications were also analyzed (ie, sulfonylureas, insulin, and thiazolidinedione).

**Results:**

Among 4851 esophageal cancer patients (8404 person-years), 4072 (84%) died during follow-up. Compared with esophageal cancer patients with diabetes but not using metformin, decreased all-cause mortality was indicated among nondiabetic patients (without metformin) (HR = 0.86, 95% CI = 0.77 to 0.96) and diabetic patients who used metformin (HR = 0.86, 95% CI = 0.75 to 1.00). The hazard ratios of all-cause mortality decreased with a higher daily dose of metformin (*P*_trend_ = .04). The corresponding hazard ratios for disease-specific mortality were similar but slightly attenuated. The results were also similar in separate analyses of esophageal cancer patients with adenocarcinoma or squamous cell carcinoma, with tumor stage I-II or III-IV, and in those who had or had not undergone surgery. No associations with mortality outcomes were found for use of sulfonylureas, insulin, or thiazolidinedione.

**Conclusions:**

Diabetes was associated with an increased all-cause mortality, whereas metformin use was associated with decreased all-cause mortality among esophageal cancer patients. More research is needed to determine if metformin affects survival in esophageal cancer.

Esophageal cancer is a major cancer burden globally (1). During the past 3 decades, the number of new cases and deaths in esophageal cancer has increased by 52% and 40%, respectively (2), and a further 35% increase is expected within 1 decade (3). With 0.57 million new cases and 0.51 million deaths, esophageal cancer is the seventh most common cancer and sixth leading cause of cancer-related mortality (4). Despite efforts to advance the treatment, only 10%-20% of patients survive 5 years in high-income countries (5). It is essential to identify prognostic factors. Diabetes has a high prevalence globally (20% in the age group 65-79 years) (6) and is associated with an increased mortality from some cancer types such as colon cancer (7), possibly because of reduced immunocompetence and increased general inflammation (8). Inconsistent results have been reported regarding the prognostic role of diabetes in esophageal cancer (7,9–12). Some studies have indicated that diabetes is associated with a reduced survival among esophageal cancer patients (9,11), whereas other studies did not find such association (7,12) or even reported an improved survival (10). The antidiabetic medication metformin (a biguanide) has anticancer properties and can decrease the risk of developing esophageal cancer (13), eg, by inhibiting the growth of esophageal cancer cells (14-16). A beneficial prognostic effect of metformin has been observed for some other tumors, eg, colon, lung, and prostate cancer (17,18). The few studies that have examined the association between metformin use and mortality in esophageal cancer have provided conflicting results and only evaluated surgically treated patients (19,20). No study has evaluated the prognostic role of metformin use in esophageal cancer independent of treatment.

We aimed to test the hypotheses that diabetes is associated with a worsened survival in esophageal cancer and that metformin therapy is associated with an improved survival. Therefore, we collected a large and unselected cohort of patients with esophageal cancer, explored interactions between the 2 exposures, and compared the results with the use of antidiabetic medications other than metformin.

## Methods

### Study design

This was a nationwide Swedish population-based cohort study during the study period July 1, 2005, to December 31, 2019. Data came from an updated version of the Swedish Prescribed Drugs and Health (SPREDH) cohort. The original version of SPREDH has been described in detail elsewhere (13,21). Briefly, SPREDH comprises registry data regarding cancer (Cancer Registry), diagnoses (Patient Registry), medications (Prescribed Drug Registry), and mortality (Cause of Death Registry) from the vast majority (9.1 million) of Swedish residents. These registries have excellent data quality and almost 100% completeness (13,21). All individuals with a newly diagnosed esophageal cancer in SPREDH between June 30, 2006, and December 31, 2018, were included in this study and were followed up until death or end of the study period (December 31, 2019), whichever occurred first. Study entry was the date of the esophageal cancer diagnosis (code C15 in the *International Classification of Diseases* version 10). We excluded patients with other malignancy (except for nonmelanoma skin cancer), gestational diabetes, polycystic ovarian syndrome (a rare indication for metformin therapy), and diabetes prevention (ie, metformin users without diabetes). This study was approved by the regional ethical review board in Stockholm (diary number 2016/982-31/4), and according to Swedish law, written informed consent was not required for this register-based study.

### Exposures

The esophageal cancer patients included in the cohort were classified into 3 main exposure groups based on the exposure status before study entry: 1) diabetes without metformin use, where diabetes was identified in the patient registry (*International Classification of Diseases* version 10 codes E10-E14) or by the dispensation of any antidiabetic medications, except for metformin during the year before study entry; 2) no diabetes or metformin use during the year before entry; and 3) diabetes with metformin use (ie, at least 1 dispensation of metformin during the year before entry). Use of other antidiabetic medication together with metformin was allowed. Among metformin users, the total amount of daily defined doses during the year before entry was assessed. To compare the results for metformin with other antidiabetic medications, 3 additional exposures were diabetes combined with at least 1 dispensation of 1) sulfonylurea, 2) insulin, and 3) thiazolidinedione during the year before study entry. All antidiabetic medication use was determined using the anatomical therapeutic chemical codes listed in Supplementary Table 1 (available online).

### Covariates

Six covariates were considered potential confounders: age at esophageal cancer diagnosis (continuous), sex (male or female), calendar year of esophageal cancer diagnosis (continuous), obesity (obesity-related diagnosis or bariatric surgery before study entry, yes or no), comorbidity (Charlson comorbidity index, 0, 1, or ≥2), and use of other medications (yes or no; ie, nonsteroidal anti-inflammatory medications or statins during the year before study entry). The Charlson comorbidity index included specific diseases diagnosed within 3 years before study entry, excluding esophageal cancer and diabetes (22). Three covariates were considered potential effect modifiers: histological type of esophageal cancer (adenocarcinoma or squamous cell carcinoma), clinical tumor stage (I, II, III, or IV), and surgery with esophagectomy (yes or no). These variables were considered effect modifiers because metformin use was determined before the esophageal cancer diagnosis, and there is no known association between metformin use and these variables.

### Outcomes

Outcomes were overall all-cause mortality and disease-specific mortality. All-cause mortality included all deaths after study entry (ie, date of esophageal cancer diagnosis). Disease-specific mortality was defined as esophageal cancer being a main cause of death in the Cause of Death Registry during the follow-up. Information about dates and causes of death was available until December 31, 2019.

### Statistical analysis

Multivariable Cox regression models were applied to estimate hazard ratios (HRs) with 95% confidence intervals (CIs) for the exposures in relation to the outcomes. The proportional hazard assumption was assessed with a likelihood-ratio test comparing the model with and without an interaction term between time and exposure status by diabetes and metformin use, and we confirm that the test did not show any violation of the assumption. Two models were applied: a crude model without any adjustments and a main model adjusted for the 6 confounders with categorizations presented above (covariates). The main analysis compared the 3 main exposure groups, where diabetic patients without metformin use were the reference group. Another analysis examined dose-response associations of metformin use, where the cumulative amount of metformin during the year before cancer diagnosis was divided into 3 similarly sized groups (tertiles), and the corresponding hazards of mortality were compared with the reference group (diabetes without metformin). Kaplan–Meier curves of all-cause and disease-specific mortality were computed by metformin doses using the log-rank test. To avoid immortal time bias in the dose-response analyses, the landmark method was used, where we defined the drug exposure status prior to the study entry (landmark time) (23,24). *P*_trend_ was computed. Separate analyses were conducted for the follow-up periods 0-1 year and more than 1 year. Dose-response analyses were also performed for sulfonylurea and insulin but not for thiazolidinedione because of the small number of patients.

Stratified analyses were conducted by age groups (aged younger than 65, 65-70, and older than 70 years), sex (male and female), comorbidity (Charlson comorbidity index 0, 1, and ≥2), histology (squamous cell carcinoma and adenocarcinoma), tumor stage (I-II and III-IV), and surgery with esophagectomy (yes and no). The analyses of esophagectomy were more extensive than other variables. Two sensitivity analyses were applied. First, a subcohort of new metformin users was established, where new metformin use was defined as no metformin use between July 1, 2005, and June 30, 2006, but only later. In this subcohort, 3 groups were classified according to their average cumulative dose of metformin during the year before study entry. The group with the lowest dose was used as the reference. In the second sensitivity analysis, the effect modifiers tumor histology and clinical tumor stage were added to the main multivariable model. Missing data were categorized as a separate group. A 2-sided *P* value less than .05 was considered statistically significant. The analyses followed a predefined protocol and were conducted using the statistical software STATA MP 15.0 (StataCorp LLC, TX, USA).

## Results

### Patient characteristics

The study included 4851 esophageal cancer patients who were followed up for a mean of 1.7 years, generating 8404 person-years. Of these patients, 4072 (84%) died during the follow-up. Among all patients, 852 (18%) had diabetes, and of these, 473 (56%) used metformin. Table 1 shows patient characteristics by the 3 main exposure groups (ie, diabetes without metformin, no diabetes or metformin, and diabetes with metformin). Compared with diabetic patients without metformin, nondiabetic patients or diabetic patients with metformin tended to be younger and had fewer comorbidities but had more advanced tumor stages of esophageal cancer.

**Table 1.** Characteristics of the esophageal cancer patients by status of diabetes and metformin use at study entry

Characteristic	Diabetes + no metformin (n = 379)	No diabetes + no metformin (n = 3999)	Diabetes + metformin (n = 473)
Age, mean (SD), y	72.8 (10.2)	69.5 (11.2)	70.1 (9.1)
Sex, No. (%)			
Male	294 (77.6)	2942 (73.6)	391 (82.7)
Female	85 (22.4)	1057 (26.4)	82 (17.3)
Calendar year (SD)	2012 (4)	2012 (4)	2013 (4)
Histology, No. (%)			
Adenocarcinoma	224 (59.1)	2212 (55.3)	357 (75.5)
Squamous cell carcinoma	136 (35.9)	1571 (39.3)	86 (18.2)
Others or missing	19 (5.5)	216 (5.4)	30 (6.3)
Obesity diagnosis, No. (%)	21 (5.5)	59 (1.5)	43 (9.1)
Charlson Comorbidity Index, No. (%)^a^			
0	191 (50.4)	2849 (71.2)	311 (65.8)
1	97 (25.6)	765 (19.1)	97 (20.5)
≥2	91 (24.0)	385 (9.6)	65 (13.7)
NSAIDS or statin use, No. (%)	248 (65.4)	1754 (43.9)	382 (80.8)
Tumor stage, No. (%)^b^			
I	61 (20.2)	568 (16.3)	76 (18.3)
II	35 (11.6)	498 (14.3)	42 (10.1)
III	84 (27.7)	881 (25.3)	118 (28.4)
IV	123 (40.6)	1533 (44.1)	180 (43.3)
Surgery with esophagectomy			
No	338 (89.2)	3157 (78.9)	364 (77.0)
Yes	41 (10.8)	842 (21.1)	109 (23.0)
Metformin, No. (%)			
<150 DDD	NA	NA	141 (29.8)
150-299 DDD	NA	NA	155 (32.8)
≥300 DDD	NA	NA	177 (37.4)
Mean follow-up years (SD)	1.3 (2.2)	1.8 (2.3)	1.6 (2.3)
Person-years	508.5	7122.4	773.5
Deaths from all causes	344	3350	378

a Diabetes is not included. DDD = defined daily dose; NA = not applicable; NSAIDS = nonsteroidal anti-inflammatory drugs.

b The number of patients with tumor stage information does not equal the total number of study patients because of missingness in tumor stage.

### Diabetes and mortality

Table 2 shows crude and adjusted hazard ratios for diabetes status concerning mortality among esophageal cancer patients not using metformin. Compared to diabetic patients without metformin, patients without diabetes (and no metformin) had a decreased all-cause mortality (HR = 0.86, 95% CI = 0.77 to 0.96) and a borderline decreased disease-specific mortality (HR = 0.91, 95% CI = 0.80 to 1.03). Stratified analyses showed no major differences in mortality, including separate analyses for histologic types and tumor stage (Table 3). Nondiabetic patients had no clearly decreased all-cause mortality compared with diabetic patients not using sulfonylureas (HR = 0.92, 95% CI = 0.84 to 1.16), insulin (HR = 0.94, 95% CI = 0.86 to 1.05), or thiazolidinedione (HR = 0.93, 95% CI = 0.85 to 1.01) (Table 4). Similar results were found for disease-specific mortality (Supplementary Table 2, available online). Within the first year after the esophageal cancer diagnosis, nondiabetic patients had a decreased all-cause mortality (HR = 0.83, 95% CI = 0.73 to 0.95) and disease-specific mortality (HR = 0.85, 95% CI = 0.73 to 0.98) compared with diabetic patients not using metformin (Table 2). Nondiabetic patients had a borderline decreased all-cause mortality within the first year of follow-up compared to diabetic patients not using sulfonylurea, insulin, or thiazolidinedione (Table 4). No associations were identified after a follow-up of more than 1 year for any of the antidiabetic medications (Tables 2 and 4).

**Table 2.** Disease-specific and all-cause mortality among esophageal cancer patients by diabetes status and metformin use

Patient status	Person-years	Disease-specific death	All-cause death	Disease-specific mortality		All-cause mortality	
				Crude HR (95% CI)	Adjusted HR (95% CI)^a^	Crude HR (95% CI)	Adjusted HR (95% CI)^a^
Diabetes status	8404	3287	4072				
Overall							
Diabetes without metformin	508	269	344	1.00 (Referent)	1.00 (Referent)	1.00 (Referent)	1.00 (Referent)
No diabetes	7122	2723	3350	0.78 (0.69 to 0.89)	0.91 (0.80 to 1.03)	0.75 (0.67 to 0.83)	0.86 (0.77 to 0.96)
Follow-up 0-1 year							
Diabetes without metformin	213	219	260	1.00 (Referent)	1.00 (Referent)	1.00 (Referent)	1.00 (Referent)
No diabetes	2647	1958	2301	0.72 (0.63 to 0.83)	0.85 (0.73 to 0.98)	0.72 (0.63 to 0.81)	0.83 (0.73 to 0.95)
Follow-up >1 year							
Diabetes without metformin	295	50	84	1.00 (Referent)	1.00 (Referent)	1.00 (Referent)	1.00 (Referent)
No diabetes	4475	765	1049	1.03 (0.78 to 1.38)	1.16 (0.87 to 1.54)	0.84 (0.67 to 1.05)	0.94 (0.75 to 1.18)
Metformin use							
Overall							
No	508	269	344	1.00 (Referent)	1.00 (Referent)	1.00 (Referent)	1.00 (Referent)
<150 DDD	233	92	111	0.80 (0.63 to 1.01)	0.92 (0.72 to 1.17)	0.74 (0.60 to 0.92)	0.83 (0.66 to 1.03)
150-299 DDD	223	96	128	0.76 (0.60 to 0.96)	0.92 (0.73 to 1.17)	0.80 (0.65 to 0.98)	0.94 (0.76 to 1.15)
≥300 DDD	317	107	139	0.64 (0.52 to 0.81)	0.80 (0.63 to 1.01)	0.65 (0.53 to 0.79)	0.78 (0.64 to 0.96)
*P*_trend_^b^				<.001	.07	<.001	.04
Any dose of metformin	774	295	378	0.72 (0.61 to 0.85)	0.90 (0.76 to 1.07)	0.72 (0.62 to 0.83)	0.86 (0.75 to 1.00)
Follow-up 0-1 year							
No	213	219	260	1.00 (Referent)	1.00 (Referent)	1.00 (Referent)	1.00 (Referent)
<150 DDD	88	73	85	0.81 (0.62 to 1.06)	0.94 (0.72 to 1.23)	0.80 (0.62 to 1.02)	0.89 (0.70 to 1.14)
150-299 DDD	99	73	91	0.73 (0.56 to 0.95)	0.87 (0.67 to 1.14)	0.76 (0.60 to 0.97)	0.88 (0.69 to 1.12)
≥300 DDD	127	67	83	0.53 (0.40 to 0.69)	0.66 (0.50 to 0.87)	0.55 (0.43 to 0.70)	0.67 (0.52 to 0.86)
*P*_trend_^b^				<.001	.003	<.001	.003
Any dose of metformin	315	213	259	0.66 (0.55 to 0.80)	0.84 (0.70 to 1.02)	0.68 (0.57 to 0.81)	0.83 (0.69 to 0.98)
Follow-up >1 year							
No	295	50	84	1.00 (Referent)	1.00 (Referent)	1.00 (Referent)	1.00 (Referent)
<150 DDD	145	19	26	0.76 (0.45 to 1.28)	0.87 (0.51 to 1.48)	0.62 (0.40 to 0.95)	0.68 (0.44 to 1.06)
150-299 DDD	124	23	37	0.92 (0.56 to 1.51)	1.14 (0.69 to 1.88)	0.92 (0.63 to 1.36)	1.11 (0.75 to 1.65)
≥300 DDD	190	40	56	1.07 (0.71 to 1.63)	1.30 (0.85 to 1.97)	0.92 (0.66 to 1.30)	1.07 (0.76 to 1.51)
*P*_trend_^b^				.57	.16	.99	.38
Any dose of metformin	459	82	119	0.94 (0.66 to 1.34)	1.15 (0.81 to 1.64)	0.82 (0.62 to 1.09)	0.97 (0.73 to 1.28)

a Model adjusted for age, sex, calendar year, obesity, comorbidity, and other medications (nonsteroidal anti-inflammatory drugs or statin). CI = confidence interval; DDD = defined daily dose; HR = hazard ratio.

b Modeled by taking the mean of the DDD quantity as 0, 75, 203, and 403 DDD.

**Table 3.** Stratified analyses of disease-specific and all-cause mortality among esophageal cancer patients

Characteristics	Person-years	Disease-specific death	All-cause death	Disease-specific mortality			All-cause mortality		
				HR (95% CI)			HR (95% CI)		
				Diabetes + no metformin	No diabetes + no metformin^a^	Diabetes+ metformin^a^	Diabetes + no metformin	No diabetes + no metformin^a^	Diabetes + metformin^a^
Age, y									
<65	3481	909	1127	1.00 (Referent)	0.74 (0.55 to 0.99)	0.76 (0.53 to 1.10)	1.00 (Referent)	0.70 (0.54 to 0.90)	0.70 (0.51 to 0.96)
65-70	2048	698	867	1.00 (Referent)	0.94 (0.71 to 1.24)	0.99 (0.70 to 1.40)	1.00 (Referent)	0.94 (0.73 to 1.21)	1.06 (0.77 to 1.44)
>70	2876	1680	2078	1.00 (Referent)	0.94 (0.80 to 1.11)	0.90 (0.72 to 1.13)	1.00 (Referent)	0.88 (0.76 to 1.01)	0.84 (0.69 to 1.03)
Sex									
Male	6381	2456	3051	1.00 (Referent)	0.93 (0.81 to 1.08)	0.91 (0.75 to 1.09)	1.00 (Referent)	0.89 (0.78 to 1.02)	0.87 (0.73 to 1.02)
Female	2023	831	1021	1.00 (Referent)	0.79 (0.60 to 1.03)	0.86 (0.59 to 1.24)	1.00 (Referent)	0.73 (0.57 to 0.92)	0.81 (0.59 to 1.13)
Comorbidity									
0	6454	2219	2732	1.00 (Referent)	0.87 (0.73 to 1.05)	0.89 (0.71 to 1.11)	1.00 (Referent)	0.85 (0.72 to 0.99)	0.85 (0.70 to 1.04)
1	1363	672	839	1.00 (Referent)	1.06 (0.82 to 1.37)	1.00 (0.70 to 1.43)	1.00 (Referent)	1.00 (0.80 to 1.25)	1.00 (0.73 to 1.36)
≥2	587	396	501	1.00 (Referent)	0.78 (0.60 to 1.03)	0.74 (0.50 to 1.09)	1.00 (Referent)	0.72 (0.56 to 0.91)	0.72 (0.51 to 1.01)
Histology									
EAD	5600	1955	2529	1.00 (Referent)	0.88 (0.75 to 1.04)	0.91 (0.74 to 1.11)	1.00 (Referent)	0.86 (0.75 to 0.99)	0.88 (0.74 to 1.05)
ESCC	2805	1332	1543	1.00 (Referent)	0.95 (0.77 to 1.17)	1.01 (0.74 to 1.39)	1.00 (Referent)	0.84 (0.70 to 1.01)	0.85 (0.64 to 1.14)
Stage									
I-II	3837	642	871	1.00 (Referent)	0.98 (0.72 to 1.32)	0.92 (0.61 to 1.37)	1.00 (Referent)	0.78 (0.62 to 0.99)	0.83 (0.60 to 1.16)
III-IV	4568	2645	3201	1.00 (Referent)	0.91 (0.79 to 1.05)	0.90 (0.75 to 1.08)	1.00 (Referent)	0.90 (0.79 to 1.02)	0.86 (0.74 to 1.03)

a Model adjusted for age, sex, calendar year, obesity, comorbidity, and other medications (nonsteroidal anti-inflammatory drugs or statin). CI = confidence interval; EAD = esophageal adenocarcinoma; ESCC = esophageal squamous cell carcinoma; HR = hazard ratio.

**Table 4.** All-cause mortality among esophageal cancer patients by use of sulfonylureas, insulin, and thiazolidinedione

Patient status	Sulfonylureas			Insulin			Thiazolidinedione		
	Person-years	No. of deaths	HR (95% CI)^a^	Person-years	No. of deaths	HR (95% CI)^a^	Person-years	No. of deaths	HR (95% CI)^a^
Diabetes status									
Overall									
Diabetes without index medication^b^	1068	604	1.00 (Referent)	830	443	1.00 (Referent)	1244	706	1.00 (Referent)
No diabetes	7122	3350	0.92 (0.84 to 1.16)	7122	3350	0.94 (0.86 to 1.05)	7122	3350	0.93 (0.85 to 1.01)
Follow-up 0-1 year									
Diabetes without index medication	441	434	1.00 (Referent)	327	319	1.00 (Referent)	516	510	1.00 (Referent)
No diabetes	2647	2301	0.91 (0.82 to 1.02)	2647	2301	0.92 (0.81 to 1.03)	2647	2301	0.92 (0.83 to 1.01)
Follow-up >1 year									
Diabetes without index medication	627	170	1.00 (Referent)	503	124	1.00 (Referent)	728	196	1.00 (Referent)
No diabetes	4475	1049	0.94 (0.80 to 1.11)	4475	1049	1.94 (0.85 to 1.24)	4475	1049	0.96 (0.82 to 1.12)
Use of the index medication^b^									
Overall									
None	1068	604	1.00 (Referent)	830	443	1.00 (Referent)	1244	706	1.00 (Referent)
Lower third dose	74	39	0.94 (0.68 to 1.30)	123	84	1.00 (0.79 to 1.26)	NA	NA	NA
Middle third dose	61	36	0.92 (0.66 to 1.29)	153	106	1.12 (0.90 to 1.39)	NA	NA	NA
Upper third dose	79	43	0.99 (0.72 to 1.35)	176	89	1.07 (0.85 to 1.35)	NA	NA	NA
*P*_trend_^c^			.74			.41			NA
Any dose of index medication	214	118	0.95 (0.78 to 1.16)	452	279	1.06 (0.91 to 1.23)	38	16	1.01 (0.62 to 1.67)
Follow-up 0-1 year									
None	441	435	1.00 (Referent)	327	319	1.00 (Referent)	516	510	1.00 (Referent)
Lower third dose	26	27	0.96 (0.65 to 1.41)	65	59	0.87 (0.66 to 1.16)	NA	NA	NA
Middle third dose	27	25	0.88 (0.59 to 1.32)	71	76	1.03 (0.80 to 1.32)	NA	NA	NA
Upper third dose	33	33	1.07 (0.75 to 1.52)	65	65	1.09 (0.83 to 1.43)	NA	NA	NA
*P*_trend_^c^			.88			.50			NA
Any dose of index medication	86	85	0.97 (0.77 to 1.22)	201	200	1.00 (0.83 to 1.19)	12	9	0.94 (0.48 to 1.81)
Follow-up >1 year									
None	626	170	1.00 (Referent)	503	124	1.00 (Referent)	728	196	1.00 (Referent)
Lower third dose	48	12	0.91 (0.50 to 1.64)	58	25	1.44 (0.93 to 2.22)	NA	NA	NA
Middle third dose	33	11	0.98 (0.53 to 1.80)	82	30	1.42 (0.95 to 2.12)	NA	NA	NA
Upper third dose	46	10	0.76 (0.40 to 1.44)	111	24	1.02 (0.65 to 1.58)	NA	NA	NA
*P*_trend_^c^			.40			.65			NA
Any dose of index medication	128	33	0.91 (0.63 to 1.32)	251	79	1.23 (0.93 to 1.63)	26	7	1.15 (0.54 to 2.44)

a Model adjusted for age, sex, calendar year, obesity, comorbidity, and other medications (nonsteroidal anti-inflammatory drugs or statin). CI = confidence interval; DDD = defined daily dose; HR = hazard ratio; NA = not applicable.

b Index medication indicates for each of the 3 medications (sulfonylureas, insulin, or thiazolidinedione); metformin use was possible.

c For sulfonylureas, lower third dose denotes less than 135 DDD, middle third dose denotes 135-225 DDD, and upper third dose denotes at least 225 DDD; for insulin, lower third dose denotes less than 250 DDD, middle third dose denotes 250-500 DDD, and upper third dose denotes more than 500 DDD.

Table 5 shows analyses stratified by esophagectomy for esophageal cancer. In the nonoperated group, absence of diabetes was associated with a moderate reduction in all-cause mortality (HR = 0.89, 95% CI = 0.79 to 1.00) but little apparent effect on disease-specific mortality (HR = 0.94, 95% CI = 0.82 to 1.07). No associations were found for patients who underwent esophagectomy.

**Table 5.** Disease-specific and all-cause mortality among esophageal cancer patients by surgical status

Outcomes	No surgery			Surgery with esophagectomy^a^		
	Person-years	No. of deaths	HR (95% CI)^b^	Person-years	No. of deaths	HR (95% CI)^b^
Disease-specific mortality						
Full cohort						
Diabetes without metformin	374	250	1.00 (Referent)	161	19	1.00 (Referent)
No diabetes	4191	2320	0.94 (0.82 to 1.07)	3546	403	0.92 (0.58 to 1.48)
Diabetes with metformin	460	246	0.90 (0.76 to 1.08)	395	49	1.08 (0.63 to 1.86)
Dose-response analysis of metformin use						
None	374	250	1.00 (Referent)	161	19	1.00 (Referent)
<150 DDD	161	80	0.89 (0.69 to 1.14)	95	12	1.13 (0.52 to 2.43)
150-299 DDD	114	84	0.96 (0.74 to 1.23)	133	12	0.99 (0.44 to 2.20)
≥300 DDD	185	82	0.75 (0.58 to 0.97)	166	25	1.34 (0.71 to 2.52)
All-cause mortality						
Full cohort						
Diabetes without metformin	374	318	1.00 (Referent)	135	26	1.00 (Referent)
No diabetes	4191	3455	0.89 (0.79 to 1.00)	2931	527	0.92 (0.62 to 1.38)
Diabetes with metformin	460	314	0.87 (0.75 to 1.02)	314	64	1.04 (0.65 to 1.65)
Dose-response analysis of metformin use						
None	374	318	1.00 (Referent)	135	26	1.00 (Referent)
<150 DDD	161	96	0.80 (0.63 to 1.01)	72	15	1.06 (0.53 to 2.15)
150-299 DDD	114	111	0.99 (0.79 to 1.24)	109	17	0.91 (0.46 to 1.81)
≥300 DDD	185	107	0.75 (0.60 to 0.94)	132	32	1.30 (0.75 to 2.26)

a Entry at the time of surgery with esophagectomy. CI = confidence interval; DDD = defined daily dose; HR = hazard ratio.

b Model adjusted for age, sex, calendar year, obesity, comorbidity, and other medications (nonsteroidal anti-inflammatory drugs or statin).

### Metformin use and mortality

Analyses of diabetic patients with metformin use are shown in Table 2. Compared with diabetic patients without metformin, diabetic patients using metformin (any dose) were associated with a decreased all-cause mortality (HR = 0.86, 95% CI = 0.75 to 1.00) but not disease-specific mortality (HR = 0.90, 95% CI = 0.76 to 1.07). The risk decreased with higher doses of metformin (*P*_trend_ = .04 for all-cause mortality and *P*_trend_ = .07 for disease-specific mortality), and the Kaplan–Meier curves showed statistical differences in survival estimates for both all-cause and disease-specific mortality (*P*_log-rank_ < .001 for both; Supplementary Figure 1, available online). The mortality was particularly decreased in a dose-response manner among metformin users during the first year of follow-up (*P*_trend_ = .003 for both all-cause and disease-specific mortality). No associations between metformin use and mortality were found for patients who survived the first year (Table 2).

The sensitivity analysis of new metformin users showed a marginally decreased all-cause and disease-specific mortality in association with a higher dose of metformin use (Supplementary Table 3, available online). The sensitivity analyses with further adjustment for tumor histology and stage showed similar results as in the main analyses (Supplementary Table 4, available online). The use of other antidiabetic medications than metformin was not associated with the all-cause mortality (Table 4) or disease-specific mortality (Supplementary Table 2, available online). A higher dose of metformin (more than 300 daily defined dose) suggested a further decreased all-cause and disease-specific mortality in nonoperated patients, whereas no such association was found in the esophagectomy group (Table 5).

## Discussion

This study suggests that diabetes was associated with an increased all-cause mortality among esophageal cancer patients, whereas the use of metformin was related to a lower all-cause mortality, and the association had a dose-dependent appearance. The results for esophageal cancer–specific mortality were similar but slightly attenuated. A decreased all-cause mortality in association with metformin use was particularly observed in nonoperated patients. However, the use of other antidiabetic medications (ie, sulfonylurea, insulin, or thiazolidinedione) was not associated with mortality in this study.

This is the first study examining how diabetes and metformin use were separately associated with the mortality in esophageal cancer independent of treatment. The study benefits from a large sample of esophageal cancer patients, nationwide and population-based cohort design, and long and complete follow-up. Selection bias and information bias should be negligible owing to the completeness and accuracy of the data in the national registries. Yet, this study is limited by possible unmeasured confounding from, for example, measured body mass index, tobacco smoking, alcohol consumption, and measures of metabolic syndrome or glycemic control, that might influence mortality. However, some of the confounding were counteracted by the adjustment for several relevant covariates, including obesity and other comorbidities. This study was unable to distinguish the possible effects of metformin from the possible effects of other diabetic medications. Analyses accounting for other antidiabetic medications did not show any associations with all-cause mortality or disease-specific mortality, raising the hypothesis that reduced mortality might be associated specifically with the use of metformin rather than with diabetes or with the use of other diabetic medications. Heterogeneity of participants with different diabetes may exist in this study, and stratification analyses by subtypes of diabetes could not be done because some participants received the diabetic diagnosis from their family doctors, which were not included in the Patient Registry. But in Sweden, more than 90% of diabetes was type 2 diabetes, and an annually updated prescription for any medication was required and recorded in the Prescribed Drug Registry, which was used for diabetes indications (25). Therefore, misclassification should be limited in the study. The limited statistical power is a weakness in some of the subgroup analyses, not least for the analyses of the esophagectomy group, and conclusive findings cannot be drawn from these analyses. We were unable to perform analyses that were stratified for other potential confounders that were rare, such as obesity.

Diabetes has been associated with an increased mortality in patients with cancer of the colon, pancreas, breast, liver, and bladder (7). The present study indicated a moderately increased mortality in esophageal cancer patients who had diabetes but not using metformin, which is consistent with some previous publications (9,11) but not with others (7,10,12,26). The discrepancies in results may be explained by some methodological issues, ie, low statistical power (7) or confounding by metformin use (10,26). Selection of esophageal cancer patients who underwent esophagectomy did not show any increased mortality with diabetes (12), which was similar to the results in the esophagectomy subgroup in the present study. The increased mortality in esophageal cancer patients with diabetes in the present study was largely driven by nonoperated patients, which could be due to reduced immunocompetence and increased chronic inflammation associated with diabetes (8). It is also possible that some diabetic patients received less aggressive treatment for their esophageal cancer (27). Complications from diabetes and comorbidities associated with diabetes may explain the slightly stronger associations with all-cause mortality compared with disease-specific mortality (28).

The association between metformin use and a lower mortality found in this study is consistent with 2 small studies of esophageal cancer patients (19,29). In contrast, 2 other studies reported no survival benefit; one included 461 esophageal cancer patients of whom 32 diabetics used metformin and 11 did not (20), and the other compared 29 diabetic metformin users with 21 nonusers in a cohort of 285 esophageal cancer patients (30). The lack of association in these studies could be because of low statistical power. In this study, we found the survival benefit among diabetic patients who used metformin was seemingly dose dependent and appeared particularly in the first year after cancer diagnosis, which indicated that metformin may interact with or enhance the therapeutic effect by chemotherapy, radiotherapy, or surgery and could be used as an adjuvant treatment for cancer patients (31). The dose-response analysis was conducted only to assess the validity of the findings by metformin and shall not be interpreted as advocating the use of high doses of metformin, which could introduce problems with blood glucose control and toxicity. Further studies, including large randomized controlled trials, are required to confirm the associations found in the current study before any clinical implications may be considered. Preclinical studies have shown several anticancer properties of metformin of relevance for esophageal cancer. Metformin may inhibit activation of the nuclear factor kappa B, inhibit tumor growth, induce apoptosis in esophageal cancer cell lines, and inhibit cell motility by inducing E-cadherin expression (32). For esophageal adenocarcinoma, metformin has been shown to block cell cycle of G0 to G1 transition in the cell cycle, accompanied by decreased cyclin D1 levels and retinoblastoma protein phosphorylation, as well as reduced phosphorylation of the epidermal growth factor receptor, insulin-like growth factor, and angiogenesis-related proteins (33). An in vivo study of athymic nude mice bearing xenograft tumor found a reduced tumor proliferation following metformin treatment (33). A randomized clinical trial comparing metformin with chemotherapy vs chemotherapy alone among nondiabetic patients with gastrointestinal cancers found increased phosphorylation of adenosine monophosphate–activated protein kinase and showed improved disease control (34). Thus, there are biological mechanisms that may explain the association.

In conclusion, this population-based cohort study indicates that survival in esophageal cancer was negatively associated with diabetes but positively associated with metformin use and not with other antidiabetic medications (ie, sulfonylureas, insulin, or thiazolidinedione). Additional observational studies showing that use of metformin in association with lower mortality among individuals with newly diagnosed esophageal cancer would support a call for randomized trials of metformin in this setting.

## Supplementary Material

pkad043_Supplementary_Data

## Data Availability

Data underlying this publication was obtained from Statistics Sweden (scb.se), and the National Board of Health and Welfare (Socialstyrelsen, socialstyrelsen.se) and can be directly requested from these institutions. The data in summarized form are available upon request to the senior author.
