# Supplementary material for: Diabetes, metformin use, and survival in esophageal cancer: a population-based cohort study
Source: JNCI Cancer Spectr. 2023 Jun 14;7(4):pkad043. doi: 10.1093/jncics/pkad043 (PMC10322653; doi:10.1093/jncics/pkad043)
Supplement: pkad043_Supplementary_Data [file pkad043_supplementary_data.pdf]

Diabetes, metformin use and survival in esophageal cancer: a population-based cohort study

Qiao-Li Wang (MD, PhD), Giola Santoni (PhD), Jesper Lagergren (MD, PhD)

Online Supplemental Material

The following Supplementary Tables 1-4 and Supplementary Figure 1 are included in the Online Supplemental Material.

Supplementary Table 1. Anatomical therapeutic chemical (ATC) codes for the antidiabetic medications in the study

Supplementary Table 2. Risk of disease-specific mortality among esophageal cancer patients by use of sulfonylureas, insulin, and thiazolidinedione

Supplementary Table 3. Risk of disease-specific and all-cause mortality in new metformin users of esophageal cancer patients

Supplementary Table 4. Risk of disease-specific and all-cause mortality among esophageal cancer patients by metformin use, with further adjustment for tumor histology and stage

Supplementary Figure 1. Kaplan-Meier survival estimates of all-cause mortality (A) and disease-specific mortality (B) among oesophageal cancer patients according to levels of metformin use.

Supplementary Table 1. Anatomical therapeutic chemical (ATC) codes for the antidiabetic medications in the study

Name of the Glucose-lowering medications	Anatomical Therapeutic Chemical (ATC) code
Metformin and metformin-combined medication	A10BA02 A10BD02 A10BD03 A10BD05 A10BD07 A10BD08 A10BD10 A10BD11 A10BD13 A10BD14 A10BD15 A10BD16 A10BD17 A10BD18 A10BD20 A10BD22 A10BD23 A10BD25
Sulfonylureas	A10BB A10BD01 A10BD04 A10BD06
Insulin	A10A
Thiazolidinedione	A10BG A10BD04 A10BD06 A10BD09 A10BD12

Notes: For codes started with A10BD, they are combinations of two different types of anti-diabetic medications

Supplementary Table 2. Risk of disease-specific mortality among esophageal cancer patients by use of sulfonylureas, insulin, and thiazolidinedione

Antidiabetic medication	Sulfonylureas			Insulin			Thiazolidinedione		
	Person-years	Number of deaths	HR (95%CI)[a]	Person-years	Number of deaths	HR (95%CI)[a]	Person-years	Number of deaths	HR (95%CI)[a]
**Diabetes status**									
Overall									
Diabetes without index medication[b]	1068	471	1.00 (Reference)	830	346	1.00 (Reference)	1244	550	1.00 (Reference)
No diabetes	7122	2723	0.95 (0.86-1.05)	7122	2723	0.97 (0.87-1.09)	7122	2723	0.96 (0.87-1.05)
Follow-up 0-1 year									
Diabetes without index medication	441	361	1.00 (Reference)	327	265	1.00 (Reference)	516	424	1.00 (Reference)
No diabetes	2647	1958	0.92 (0.82-1.03)	2647	1958	0.92 (0.81-1.05)	2647	1958	0.92 (0.83-1.03)
Follow-up >1 year									
Diabetes without index medication	627	110	1.00 (Reference)	503	81	1.00 (Reference)	728	126	1.00 (Reference)
No diabetes	4475	765	1.05 (0.86-1.28)	4475	765	1.14 (0.90-1.43)	4475	765	1.08 (0.89-1.31)
**Use of the index medication[b]**									
Overall									
None	1068	471	1.00 (Reference)	830	346	1.00 (Reference)	1244	550	1.00 (Reference)
Lower third dose	74	32	0.96 (0.67-1.39)	123	63	0.96 (0.73-1.25)	NA	NA	NA
Middle third dose	61	31	1.01 (0.70-1.45)	153	87	1.15 (0.90-1.46)	NA	NA	NA
Upper third dose	79	30	0.89 (0.62-1.29)	176	68	1.04 (0.80-1.35)	NA	NA	NA
P for trend[c]			0.49			0.57			NA
Any dose of index medication	214	93	0.94 (0.75-1.18)	452	218	1.05 (0.88-1.24)	38	14	1.10 (0.64-1.87)
Follow-up 0-1 year									
None	441	361	1.00 (Reference)	327	265	1.00 (Reference)	516	424	1.00 (Reference)
Lower third dose	26	24	0.98 (0.65-1.48)	65	47	0.86 (0.63-1.18)	NA	NA	NA
Middle third dose	27	23	0.97 (0.63-1.47)	71	66	1.07 (0.81-1.40)	NA	NA	NA
Upper third dose	33	24	0.92 (0.61-1.39)	65	54	1.08 (0.80-1.46)	NA	NA	NA
P for trend[c]			0.66			0.55			NA
Any dose of index medication	86	71	0.95 (0.73-1.22)	201	167	1.00 (0.82-1.21)	12	8	0.93 (0.46-1.87)
Follow-up >1 year									
None	627	110	1.00 (Reference)	503	81	1.00 (Reference)	728	126	1.00 (Reference)
Lower third dose	48	8	0.92 (0.44-1.89)	58	16	1.34 (0.78-2.30)	NA	NA	NA
Middle third dose	33	8	1.08 (0.53-2.22)	82	21	1.48 (0.91-2.40)	NA	NA	NA
Upper third dose	46	6	0.74 (0.32-1.69)	111	14	0.92 (0.52-1.62)	NA	NA	NA
P for trend[c]			0.53			0.91			NA

Any dose of index medication	128	22	0.93 (0.59-1.46)	251	51	1.21 (0.85-1.72)	26	6	1.49 (0.66-3.40)

[a] Model adjusted for age, sex, calendar year, obesity, comorbidity, and other medications (non-steroidal anti-inflammatory drugs or statin)

[b] Index medication indicates each of the three mentioned medications in column (sulfonylureas, insulin, or thiazolidinedione)

[c] For sulfonylureas, lower third dose denotes <135 DDD, middle third dose denotes 135-225 DDD, and upper third dose denotes ≥ 225 DDD; for insulin, lower third dose denotes <250 DDD, middle third dose denotes 250-500 DDD, and upper third dose denotes > 500 DDD

Abbreviation: HR, hazard ratio; CI, confidence interval; DDD, defined daily dose; NA, not applicable

Supplementary Table 3. Risk of disease-specific and all-cause mortality in new metformin users of esophageal cancer patients

New metformin users	Number of patients	Person-years	Number of deaths	Disease-specific mortality HR (95%CI)[a]	Number of deaths	All-cause mortality HR (95%CI)[a]
Overall	447	680	279		356	
<150 DDD	133	205	88	1.00 (Reference)	104	1.00 (Reference)
150-299 DDD	148	208	91	0.94 (0.70-1.26)	122	1.08 (0.83-1.40)
≥300 DDD	166	267	100	0.85 (0.63-1.14)	130	0.94 (0.72-1.22)
Follow-up 0-1 year						
<150 DDD	133	82	71	1.00 (Reference)	83	1.00 (Reference)
150-299 DDD	148	96	68	0.86 (0.62-1.20)	85	0.92 (0.68-1.25)
≥300 DDD	166	119	64	0.69 (0.49-0.98)	80	0.74 (0.54-1.01)
Follow-up >1 year						
<150 DDD	50	123	17	1.00 (Reference)	21	1.00 (Reference)
150-299 DDD	63	112	23	1.25 (0.66-2.35)	37	1.70 (0.99-2.92)
≥300 DDD	86	149	36	1.46 (0.81-2.61)	50	1.68 (1.00-2.81)

[a] Model adjusted for age, sex, calendar year, obesity, comorbidity, and other medications (non-steroidal anti-inflammatory drugs or statin)

Abbreviation: HR, hazard ratio; CI, confidence interval; DDD, defined daily dose

Supplementary Table 4. Risk of disease-specific and all-cause mortality among esophageal cancer patients by metformin use, with further adjustment for tumor histology and stage

	Disease-specific mortality			All-cause mortality		
	**Person-years**	**Number of deaths**	**HR (95%CI)[a]**	**Person-years**	**Number of deaths**	**HR (95% CI)[a]**
**Diabetes status**	8404	3287		8404	4072	
Overall						
Diabetes without metformin	508	269	1.00 (Reference)	508	344	1.00 (Reference)
No Diabetes	7122	2723	0.93 (0.81-1.08)	7122	3350	0.88 (0.77-1.00)
Follow-up 0-1 year						
Diabetes without metformin	213	219	1.00 (Reference)	213	260	1.00 (Reference)
No Diabetes	2647	1958	0.87 (0.74-1.02)	2647	2301	0.84 (0.73-0.98)
Follow-up >1 year						
Diabetes without metformin	295	50	1.00 (Reference)	295	84	1.00 (Reference)
No Diabetes	4475	765	1.21 (0.88-1.66)	4475	1049	0.99 (0.77-1.27)
**Metformin use**						
Overall						
None	508	269	1.00 (Reference)	508	344	1.00 (Reference)
<150 DDD	233	92	0.96 (0.74-1.24)	233	111	0.81 (0.63-1.02)
150-299 DDD	223	96	0.94 (0.72-1.22)	223	128	1.01 (0.80-1.28)
≥300 DDD	317	107	0.77 (0.59-0.99)	317	139	0.82 (0.66-1.04)
P for trend[b]			0.19			0.21
Any dose of metformin	774	295	0.94 (0.78-1.13)	774	378	0.87 (0.74-1.03)
Follow-up 0-1 year						
None	213	219	1.00 (Reference)	213	260	1.00 (Reference)
<150 DDD	88	73	1.00 (0.75-1.33)	88	85	0.89 (0.68-1.16)
150-299 DDD	99	73	0.96 (0.71-1.30)	99	91	0.92 (0.70-1.21)
≥300 DDD	127	67	0.71 (0.52-0.97)	127	83	0.70 (0.53-0.92)
P for trend[b]			0.02			0.02
Any dose of metformin	315	213	0.90 (0.73-1.11)	315	259	0.83 (0.69-1.01)
Follow-up >1 year						
None	295	50	1.00 (Reference)	295	84	1.00 (Reference)
<150 DDD	145	19	0.79 (0.45-1.40)	145	26	0.61 (0.38-1.00)
150-299 DDD	124	23	1.31 (0.77-2.23)	124	37	1.30 (0.86-1.98)
≥300 DDD	190	40	1.28 (0.80-2.04)	190	56	1.16 (0.80-1.70)
P for trend[b]			0.21			0.16
Any dose of metformin	459	82	1.12 (0.76-1.64)	459	119	0.98 (0.72-1.34)

[a] Model adjusted for age, sex, calendar year, obesity, comorbidity, other medications (non-steroidal anti-inflammatory drugs or statin), tumor histology, and stage

[b] Modeled by taking the mean of the DDD quantity as 0, 75, 203, and 403 DDD

Abbreviation: HR, hazard ratio; CI, confidence interval; DDD, defined daily dose

Supplementary Figure 1. Kaplan-Meier survival estimates of all-cause mortality (A) and disease-specific mortality (B) among esophageal cancer patients according to levels of metformin use

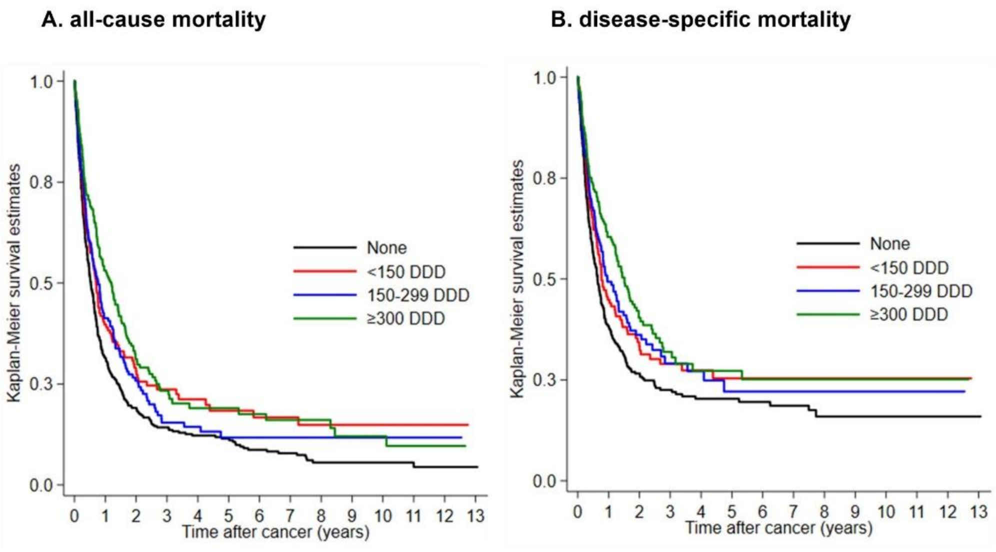

Note: $P_{\text{log-rank}}$ <0.001 for both all-cause mortality and disease-specific mortality.
